# Sansoninto attenuates aggressive behavior and increases levels of homovanillic acid, a dopamine metabolite, in social isolation-reared mice

**DOI:** 10.1016/j.jtcme.2021.08.006

**Published:** 2021-08-10

**Authors:** Takuya Watanabe, Hikari Iba, Hiroshi Moriyama, Kaori Kubota, Shutaro Katsurabayashi, Katsunori Iwasaki

**Affiliations:** a Department of Neuropharmacology, Faculty of Pharmaceutical Sciences, Fukuoka University, 8-19-1 Nanakuma, Jonan-ku, Fukuoka, 814-0180, Japan; b A.I.G. Collaborative Research Institute for Aging and Brain Sciences, Fukuoka University, 8-19-1 Nanakuma, Jonan-ku, Fukuoka, 814-0180, Japan

**Keywords:** Herbal medicine, Aggression, Hypothalamus, Novel environment, Catechol-O-methyltransferase, SST, Sansoninto, SI, social isolation, MAO, monoamine oxidase, COMT, catechol-O-methyltransferase

## Abstract

**Background and aim:**

Early-life stress is thought to affect aggressive behavior in humans and rodents. Laboratory experiments have demonstrated that Sansoninto (SST; 酸棗仁湯 suān zǎo rén tāng), a traditional herbal medicine, attenuates stress-induced abnormal behavior in rodents. However, it is unknown whether SST attenuates stress-induced aggressive behavior. The current study examined the effects of SST on aggressive behavior of mice who suffered from social isolation (SI) stress in adolescence.

**Experimental procedure:**

Five-week old mice were socially isolated for 6 weeks, and SST administration was started at 4 weeks after starting SI. Aggressive behavior and locomotor activity were examined in SST-treated mice. The content of dopamine and its metabolites in the hypothalamus were examined using high-performance liquid chromatography analysis. Gene expression analyses of monoamine oxidase-B (MAO-B), catechol-O-methyltransferase (COMT), and tyrosine hydroxylase in the hypothalamus were performed using quantitative reverse transcription polymerase chain reaction.

**Results and conclusion:**

SST attenuated SI-induced aggressive behavior and increased levels of homovanillic acid, a metabolite of dopamine. However, SST did not affect dopamine levels. SI enhanced locomotion in a novel environment and increased COMT mRNA levels. In contrast, SST-treated mice showed no significant enhancement of locomotion. SST attenuated the increase in COMT mRNA levels. Given that the dopaminergic system has been implicated in aggressive behavior, these findings suggest that SST toned down dopaminergic signaling, resulting in amelioration of aggression. SST may be useful for treatment of aggressive behavior in patients with neurotic symptoms.

## Highlight

● Sansoninto prevented increased aggressive behavior in social isolation-reared mice.
● Sansoninto attenuated increased COMT mRNA in social isolation-reared mice.
● Hypothalamic homovanillic acid level was increased by Sansoninto.

## Graphical abstract

**Figure:**
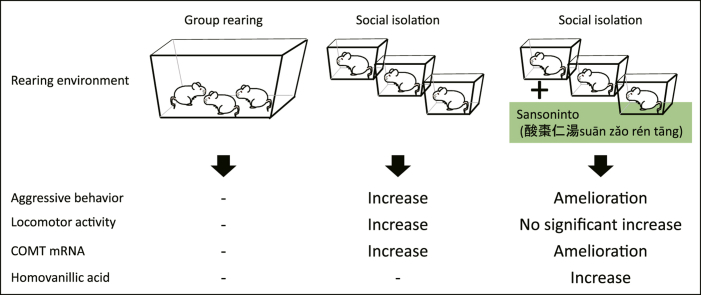

## 1 Introduction

Sansoninto (SST; 酸棗仁湯suān zǎo rén tāng) is a traditional herbal medicine prescribed for patients with weakness and fatigue, annoyance, insomnia, and/or neurotic symptoms.^1^ In preclinical research, SST was found to reverse shortened sodium pentobarbital-induced sleep in repeated cold stress conditions.^2^ SST has also been found to ameliorate abnormal behaviors induced by social isolation (SI) stress.^3^,^4^ Given that SST improves stress-induced abnormal behavior in rodents, SST may ameliorate stress-induced psychological symptoms.

SI stress induces behavioral abnormalities, including hyperactivity, attention-deficit-like behavior, aggressive behavior and sleep disorder.^5^, ^6^, ^7^, ^8^, ^9^, ^10^ SST has been reported to ameliorate attention-deficit-like behavior, hyperactivity and sleep disorder.^3^^,^^4^ However, the effects of SST on aggressive behavior are unknown.

The dopaminergic system has been implicated in aggressive behavior. Electrical stimulation of the ventral tegmentum, which is the source of dopaminergic innervation of the forebrain, is reported to facilitate defensive rage.^11^ In addition, dopamine (DA) receptor agonists have been found to decrease the threshold for hypothalamically-elicited defensive rage.^12^ A DA D2 receptor antagonist was found to suppress aggressive behavior induced by adolescent anabolic-androgenic steroid exposure.^13^ Thus, DA signaling in the hypothalamus modulates aggressive behavior.

Early-life stress in humans and rodents has been suggested to affect aggressive behavior.^14^ The present study examined the effects of SST on aggressive behavior in mice that suffered from SI stress in adolescence. To investigate the potential anti-aggression mechanisms of SST, we analyzed mRNA levels of the key metabolic enzymes of dopamine, monoamine oxidase-B (MAO-B) and catechol-O-methyltransferase (COMT), in the hypothalamus. Furthermore, levels of DA and its metabolites in the hypothalamus were analyzed.

## 2 Materials and methods

### 2.1 Animals

Male ddY mice (4 weeks old) were supplied by Japan SLC, Inc. (Shizuoka, Japan). After habituation for 1 week, mice were housed individually in cages (136 × 208 × 115 mm) for 6 weeks prior to testing. Group-reared controls were housed with 3–5 mice per cage (182 × 260 × 128 mm). In experiment 1, 62 mice were randomly divided into four groups (Group, n = 16; Vehicle, n = 15; SST300, n = 16; SST1000, n = 15; three experiments). In experiment 2, 61 mice were randomly divided into three groups (Group, n = 17; Vehicle, n = 23; SST1000, n = 21; four experiments). After the aggression test, 46 and 15 mice were sacrificed for HPLC and MAO-B activity analysis, respectively. Naïve male mice (5 weeks old) used for the aggression test were also supplied by Japan SLC, Inc. and were group-housed for 1 week prior to the test. All mice were housed at a temperature of 23 ± 2 °C with a relative humidity of 60 ± 10% under a 12 h light-dark cycle (lights on 07:00–19:00). Food and water were available ad libitum. All procedures regarding animal care and use were carried out in accord with the regulations dictated by the Experimental Animal Care and Use Committee of Fukuoka University (#1507852, #1704040).

### 2.2 Drugs

Dry powdered extracts of SST (Lot. No. 2150103010) were provided by Tsumura & Co. (Tokyo, Japan). SST comprises five dried extracts as follows: 10.0 parts Zizyphi Semen (酸棗仁 suān zǎo rén), 5.0 parts Poria (茯苓 fú líng), 3.0 parts Cnidii Rhizoma (川芎 chuān xiōng), 3.0 parts Anemarrhenae Rhizoma (知母 zhī mǔ) and 1.0 parts Glycyrrhizae radix (甘草 gān cǎo). Each plant sample was authenticated by identification of the external morphology and marker compounds of plant specimens according to the methods of the Japanese Pharmacopoeia and the standards of Tsumura & Co. The five medicinal herbs were extracted with purified water at 95 °C for 1 h, and the extraction solution was separated from the insoluble waste and concentrated by removing water under reduced pressure. Spray drying was used to produce a dried extract powder. The yield of SST was 14.8%. The quality was standardized in keeping with the Good Manufacturing Practices defined by the Japanese Ministry of Health, Labour and Welfare. The components of SST extract were previously confirmed using three-dimensional high-performance liquid chromatography (HPLC) analysis.^15^

### 2.3 Measurement of locomotor activity

Mice were individually housed in the measurement cage (345 × 403 × 177 mm), which contained an infrared detection sensor in the upper part (NS-AS01, Neuroscience Inc. Tokyo, Japan). Activity was continuously monitored with a computer using the Animal Spontaneous Activity Measurement System interface (ACT1-08, LabDesign, Ibaraki, Japan) and analyzed using analysis software (ACT-1 Light, version 1.22, LabDesign). Locomotor activity was measured for 4 h in each 1 h period.

### 2.4 Aggression test in a neutral area

The aggression test was performed according to a procedure described previously.^16^ Each SI- or group-reared mouse was placed individually in a neutral area (182 × 260 × 128 mm) 1 h before a naïve male mouse was introduced into the area as a stimulus. SI- or group-reared mouse were dye-marked on the tail for purposes of identification. The number of attacks (biting and wrestling) against the naïve mouse was video recorded for 10 min and analyzed by two observers blinded to the experimental treatment.

### 2.5 Quantitative real-time polymerase chain reaction (RT-PCR)

Quantitative RT-PCR analysis was performed in accord with a procedure described previously.^16^ The total RNA was extracted from hypothalamus. RT-PCR was conducted using primers. The sequences of the primers were as follows: 5′- CTGGGGGTTGGTGGCTATTG -3′ and 5′- CCCACTCCTTCTCTGAGCAG -3′ for COMT; 5′- ATGAGCAACAAAAGCGATGTGA -3′ and 5′- TCCTAATTGTGTAAGTCCTGCCT -3′ for MAO-B; 5′- TTGGCTGACCGCACATTT -3′ and 5′- GCCCCCAGAGATGCAAGT -3′ for tyrosine hydroxylase; and 5′-GGCTGTATTCCCCTCCATCG-3′ and 5′-CCAGTTGGTAACAATGCCATGT-3′ for β-actin.

### 2.6 DA and its metabolite content

The content of DA and its metabolites, including 3,4-dihydroxyphenylacetic acid (DOPAC) and homovanillic acid (HVA), were measured using an HPLC-electrochemical detector (HPLC-ECD) system in a manner similar to a procedure described previously.^16^ The hypothalamus was dissected, weighed and homogenized immediately after the aggression test. Following centrifugation, the supernatant was mixed with sodium acetate and filtered. The sample was then injected into the HPLC-ECD system.

### 2.7 Analysis of monoamine oxidase B activity

Monoamine oxidase B (MAO-B) activity was analyzed using Fluoro:MAO™ (Cell Technology Inc., Fremont, CA, USA) according to the manufacturer's instructions. Mice were sacrificed immediately after the aggression test. The hypothalamus was quickly dissected and homogenized with phosphate buffered saline. The protein concentration was measured using a BCA Protein Assay Reagent kit (Thermo scientific, Waltham, MA, USA). Sample solution containing 10 μg protein was mixed with a reaction cocktail. Fluorescence was detected using Centro LB960 (Berthold Technologies GmbH & Co., Bad Wildbad, Germany). MAO-B activity was expressed as % of group-reared controls.

### 2.8 Experimental procedure

**Fig. 1:**
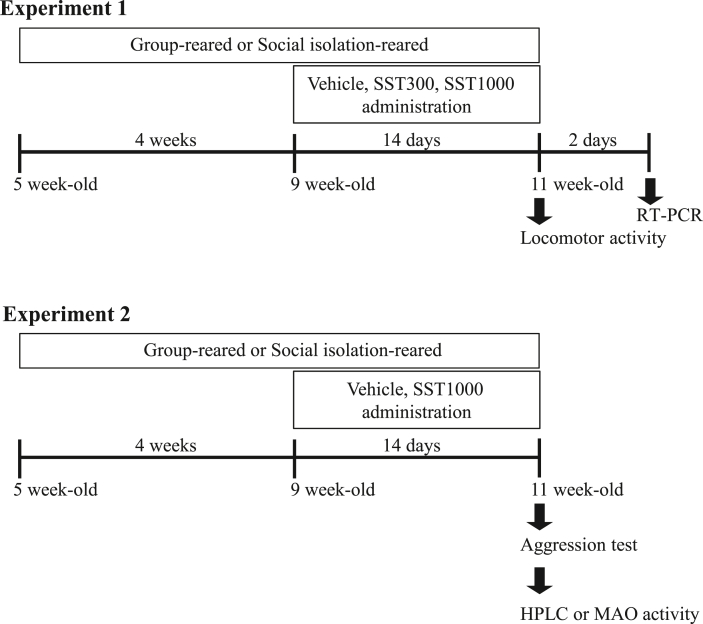
Schedule of experiments. Experiment 1 was performed for examining the effect of SST on locomotor activity. Mice (5 weeks old) were housed individually for 6 weeks (social isolation). Group-reared mice were housed with 3–5 mice/cage. Social isolation-reared mice were treated daily with distilled water (vehicle), SST300 (300 mg/kg/day) or SST1000 (1000 mg/kg/day) for 14 days before measuring locomotor activity. Mice were decapitated for examining mRNA expression 2 days after measurement of locomotor activity. Experiment 2 was performed for examining effect of SST on aggressive behavior. Social isolation-reared mice were treated daily with distilled water (vehicle) or SST1000 (1000 mg/kg/day) for 14 days before the aggression test. After the aggression test, mice were divided into two groups for HPLC analysis or MAO activity analysis. SST, Sansoninto; MAO, monoamine oxidase.

Mice were isolated for 6 weeks, and daily SST administration (300 or 1000 mg/kg/day [SST300 or SST1000], for 2 weeks, p.o.) was started at 4 weeks after starting social isolation (Fig. 1). Experiment 1: mice were placed in a cage for measuring locomotor activity on the 14th day after starting administration. 2 days after measurement of locomotor activity, mice were sacrificed to analyze mRNA expression. Experiment 2: the aggression test was performed 1 h after final administration. Immediately after the aggression test, mice were divided into two groups for analyzing DA content or MAO-B activity. For RT-PCR, HPLC, and MAO-B activity analyses, the hypothalamic area including several nuclei, such as the dorsomedial hypothalamic nucleus and lateral nucleus, was pinched off from the ventral side of the brain using forceps after the brain was dissected.

### 2.9 Statistical analysis

**Fig. 2:**
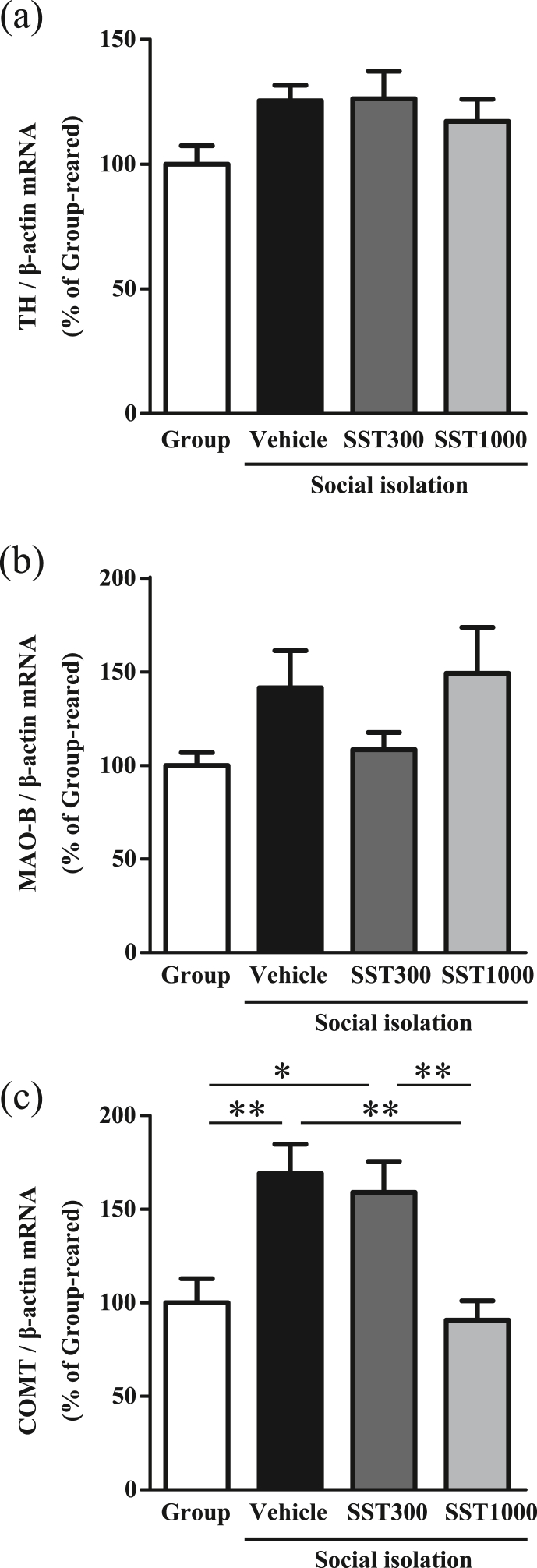
Effect of SST on expression of key enzymes in the dopaminergic system in the hypothalamus of social isolation-reared mice. Tyrosine hydroxylase (a), MAO-B (b), and COMT (c) mRNA level in the hypothalamus were examined after locomotor analysis (a and b: Group, n = 16; Vehicle, n = 15; SST300, n = 16; SST1000, n = 15; c: Group, n = 16; Vehicle, n = 13; SST300, n = 15; SST1000, n = 13). Data are expressed as % of group-reared controls. Values indicate the means ± SEM. ∗P < 0.05, ∗∗P < 0.01, significantly different.

Data for locomotor activity were evaluated for statistical significance using two-way analysis of variance (ANOVA) followed by Tukey's multiple comparisons test. Other data were evaluated for statistical significance using one-way ANOVA followed by Tukey's multiple comparisons test. Five outliers were removed from the dataset in COMT mRNA (Fig. 2c; vehicle, n = 2; SST300, n = 1; SST1000, n = 2) using the robust regression followed by outlier identification (ROUT) method (Prism 6.04 software, GraphPad, La Jolla, CA, USA). The criterion for statistical significance was P < 0.05. Data are shown as mean ± standard error (SE).

## 3 Results

### 3.1 SST1000-treated mice did not show SI-increased locomotor activity

The effect of SST on hyperactivity of SI-reared mice was examined. SST has previously been reported to demonstrate anti-depressant effects in mice at a dose of 1000 mg/kg/day.^17^ In addition, we previously reported that SST ameliorated memory impairment in dementia model rats at doses of 300 and 1000 mg/kg/day.^18^ Therefore, SST300 and SST1000 were administered in experiment 1. All four groups exhibited a time-dependent decrease of locomotor activity (two-way ANOVA: F [3, 58] = 3.013, P < 0.05), suggesting that mice were habituated to the novel environment. There were significant differences in the effects of treatment on locomotor activity (F [3, 174] = 40.85, P < 0.0001). At the first period (0–1 h), vehicle- and SST300-treated mice showed a significant increase in locomotor activity compared with group-reared mice (vehicle: P < 0.05, SST300: P < 0.05 vs. group-reared mice, Table 1). However, SST1000-treated mice did not show a significant increase in locomotor activity compared with group-reared mice. However, SST1000-treated mice also did not show a significant difference in locomotor activity compared with vehicle-treated mice. These results suggest that SI-reared mice exhibited hyperactivity in novel environments, and that SST tended to attenuate the high locomotor response to novelty in SI-reared mice.

**Table 1:** Effects of SST on hyperactivity of social isolation-reared mice.

Time (hr)	Group	Vehicle		SST300		SST1000
1	474.13 ± 78.58	781.47 ± 117.37	∗	829.56 ± 166.45	∗	613.60 ± 73.67
2	209.06 ± 46.76	284.00 ± 72.12		453.63 ± 120.28		264.33 ± 58.57
3	134.31 ± 26.76	228.40 ± 38.36		249.38 ± 82.69		258.93 ± 45.57
4	175.50 ± 47.99	290.93 ± 61.33		446.50 ± 99.95		195.00 ± 41.03

Each isolated or group-housed mouse was placed individually in a clear plastic cage, and locomotor activity was measured for 4 h in each 1 h period (Group, n = 16; Vehicle, n = 15; SST300, n = 16; SST1000, n = 15). A final administration of SST was conducted 1 h before the test. Values indicate the means ± SEM. ∗P < 0.05, vs. Group, significantly different.

### 3.2 SST-treated mice exhibited attenuation of SI-induced increase in COMT mRNA in the hypothalamus

The locomotor response to novel environments is reported to involve dopaminergic neurons in the hypothalamus.^19^ Therefore, mRNA levels of MAO-B and COMT, which are key metabolic enzymes of dopamine, and tyrosine hydroxylase, which is a rate-limiting enzyme in the biosynthesis of dopamine and other catecholamines, were examined in the hypothalamus. All four groups exhibited similar levels of tyrosine hydroxylase and MAO-B mRNA (Fig. 2a and b). Vehicle- and SST300-treated mice showed a significant increase in COMT mRNA levels compared with group-reared mice (F [3, 53] = 7.725, P < 0.001 by one-way ANOVA, vehicle: P < 0.01, SST300: P < 0.05 vs. group-reared mice using Tukey's multiple comparisons test, Fig. 2c). However, SST1000-treated mice showed a significant decrease in COMT mRNA levels compared with vehicle- and SST300-treated mice (P < 0.01 vs. vehicle- and SST300-treated mice, Fig. 2c). Contrary to our expectations, COMT mRNA levels were increased in vehicle-treated mice. In addition, SST prevented the increase in COMT mRNA. These results suggest that increase in COMT levels was caused by neuroadaptive responses to dopaminergic hyperactivity.

### 3.3 SST attenuated aggressive behavior of SI-reared mice

**Fig. 3:**
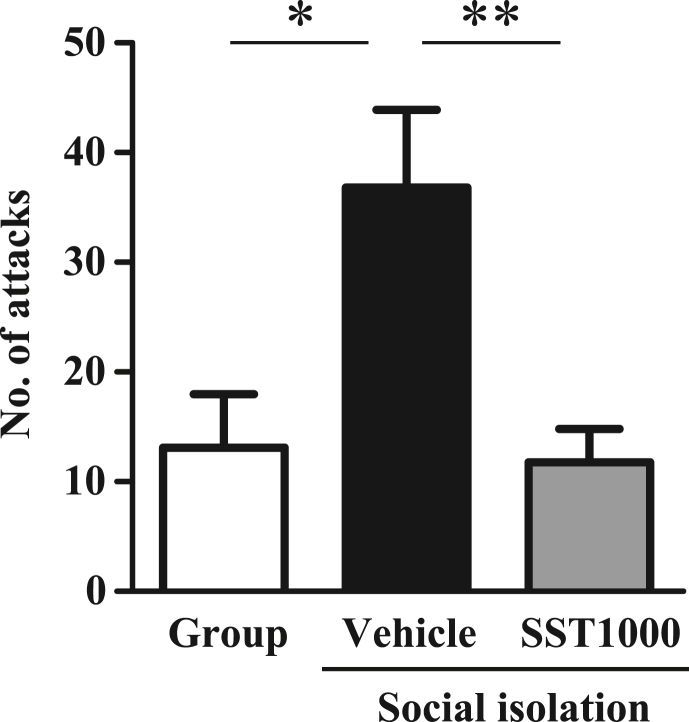
Effect of SST on aggressive behavior of social isolation-reared mice. Each isolated or group-housed mouse was placed individually in a neutral area for 1 h. Subsequently, a naïve mouse was introduced into the area. The aggressive behavior of the trial mouse against the naïve mouse was evaluated for 10 min (Group, n = 17; Vehicle, n = 23; SST1000, n = 21). A final administration of SST was conducted 1 h before the test. Values indicate the means ± SEM. ∗P < 0.05, ∗∗P < 0.01, significantly different.

The effects of SST1000 were examined in experiment 2 because SST1000, but not SST300, significantly prevented the increase in COMT levels. Vehicle-treated mice showed an increase in aggressive behavior compared with group-reared mice (F [2, 58] = 6.933, P < 0.01 by one-way ANOVA, P < 0.05 by Tukey's multiple comparisons test, Fig. 3). SST1000-treated mice showed decrease in aggressive behavior compared with vehicle-treated mice (P < 0.01 by Tukey's multiple comparisons test). These results suggest that SST1000 ameliorates SI-induced aggressive behavior.

### 3.4 SST increased content of HVA, a DA metabolite, in the hypothalamus

**Fig. 4:**
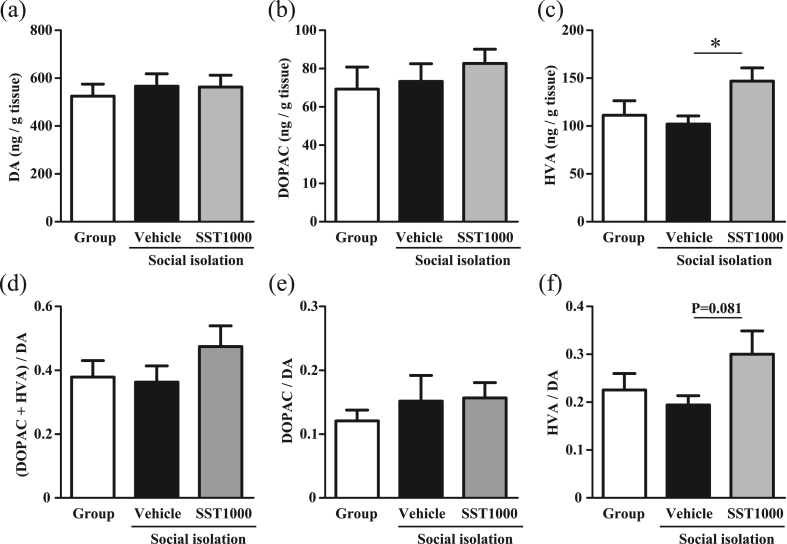
SST increased the DA metabolite HVA in the hypothalamus of social isolation-reared mice. DA (a), DOPAC (b) and HVA (c) levels in the hypothalamus were examined using HPLC after the aggression test (Group, n = 12; Vehicle, n = 18; SST1000, n = 16). Ratios of (DOPAC + HVA)/DA (d), DOPAC/DA (e), and HVA/DA (f) were analyzed. Values indicate the means ± SEM. ∗P < 0.05, significantly different.

The content of DA and its metabolites DOPAC and HVA in the hypothalamus was examined because the dopaminergic system is implicated in aggressive behavior. There were no differences in DA and DOPAC levels among all three groups (Fig. 4a and b). However, SST1000-treated mice showed higher HVA levels compared with vehicle-treated mice (F [2, 43] = 3.963, P < 0.05 by one-way ANOVA, P < 0.05 by Tukey's multiple comparisons test, Fig. 4c). The HVA/DA ratio also tended to be increased in SST1000-treated mice compared with vehicle-treated mice, although the increase was not significant (F [2, 43] = 2.519, P = 0.0923 by one-way ANOVA, P = 0.081 by Tukey's multiple comparisons test, Fig. 4f). There were no differences in (DOPAC + HVA)/DA ratio and DOPAC/DA ratio among all three groups (Fig. 4d and e). These results suggest that SST1000 affects the DA system.

### 3.5 SST did not affect MAO-B activity in the hypothalamus

To clarify the mechanisms by which SST increases HVA content, we examined the effect of SST on MAO-B activity. There were no differences in MAO-B activity among all three groups (Table 2), suggesting that SST-increased HVA content is not due to MAO-B activity.

**Table 2:** Effect of SST on MAO-B activity in hypothalamus.

	MAO-B activity (% of Group-reared)
Group	100.00 ± 16.61
Vehicle	110.19 ± 12.38
SST1000	152.40 ± 17.72

Hypothalamic MAO-B activity was examined (Group, n = 5; Vehicle, n = 5; SST1000, n = 5). Data are expressed as % of group-reared controls. Values indicate the means ± SEM.

## 4 Discussion

SST is reported to ameliorate abnormal behavior induced by cold stress and SI stress in mice.^2^, ^3^, ^4^ However, it is currently unclear whether SST ameliorates SI-induced aggressive behavior. Therefore, the present study examined the effects of SST on aggressive behavior in SI-reared mice. Because SST1000, but not SST300, attenuated the increase in COMT mRNA levels in the hypothalamus of SI-reared mice, the effect of SST1000 on aggressive behavior was investigated. The results revealed that SST ameliorated aggressive behavior and increased levels of the dopamine metabolite HVA in the hypothalamus. The present findings suggest that the DA system is involved in the SST-induced reduction in aggressive behavior.

Activation of dopamine receptor mediates aggressive behavior, which is elicited by electrical stimulation of the hypothalamus.^12^^,^^20^ In addition, D2 receptor antagonists attenuate aggressive behavior induced by SI.^21^, ^22^, ^23^ These previous reports suggest that the hypothalamic dopaminergic system plays a key role in aggressive behavior. Therefore, the present study examined the levels of DA and its metabolites in the hypothalamus. Although DA and DOPAC levels were no different among all groups, SST-treated mice showed an increase in hypothalamic HVA levels compared with vehicle-treated mice. These findings suggest that SST promotes DA metabolism, resulting in reduced dopamine receptor activation. DA is catalyzed to DOPAC by MAO-B, then DOPAC is catalyzed to HVA by COMT. However, SST did not increase MAO-B activity in the hypothalamus, suggesting that SST-increased HVA content was not due to enhancement of MAO-B activity. qPCR results also revealed no evidence that SST increased MAO-B mRNA levels. However, COMT mRNA levels were increased by SI. In addition, SST1000 attenuated the increase in COMT mRNA levels. These results regarding COMT mRNA were unexpected. Two previous studies also reported increased COMT levels in mice that showed aggressive behavior.^24^^,^^25^ COMT is localized to postsynaptic neurons.^26^ Therefore, the increase in COMT mRNA may be a neuroadaptive response to heightened and sustained dopaminergic tone. The present study was not able to clarify the mechanisms by which SST increases HVA levels. SST increased HVA levels without affecting DOPAC levels. Melatonin has also been reported to attenuate methamphetamine-induced aggression in SI-reared mice and increased HVA levels without affecting DOPAC levels.^27^ D2 receptor antagonists increase HVA levels in the brain and plasma.^28^^,^^29^ In addition, D2 receptor antagonists attenuate SI-induced aggressive behavior.^21^, ^22^, ^23^ Given that melatonin and D2 receptor antagonists affect HVA levels and aggressive behavior, some of the ingredients of SST might increase HVA levels by enhancing melatonin signaling and/or disrupting D2 receptor signaling, resulting in attenuation of aggressive behavior. SI is reported to increase the locomotor response to novelty.^30^^,^^31^ The present study also demonstrated that SI increased the locomotor response in the initial period. Locomotor responses to novel environments are implicated in dopaminergic neurons.^19^ Although SI did not increase tyrosine hydroxylase mRNA levels, COMT mRNA levels were increased by SI in the present study. Given that an overactive dopamine system has been reported in SI-reared mice,^32^ the increase in COMT mRNA levels may be caused by a neuroadaptive response. SST1000 may attenuate overactivity in the dopamine system, resulting in reduction of COMT mRNA levels and preventing an increase in the locomotor response.

Phencyclidine (PCP) is acknowledged to generate a model of schizophrenia in animals. Treatment with PCP induces hyperlocomotion in ddY mice as well as C57BL/6 mice. Furthermore, PCP treatment causes longer immobility time in ddY mice compared with C57BL/6 mice, while ICR mice do not show a PCP-induced change in the forced swim test.^33^ Social isolation has been suggested to induce more severe deficits of prepulse inhibition in ddY mice compared with C57BL/6 mice.^34^ Given that prepulse inhibition is reduced in schizophrenia patients and that PCP induces schizophrenia-like behavior, ddY mice may be vulnerable to schizophrenia-like behavior. Aggressive behavior has also been observed in schizophrenia patients.^35^ Social isolation-induced aggression has been reported in ddY mice in a number of studies.^16^^,^^36^, ^37^, ^38^ Taken together, these findings suggest that ddY mice may be a useful model for examining social isolation-induced psychosis-like behavior.

The present study involved at least one limitation. We did not examine whether agonists and antagonists of DA receptors block or enhance the effect of SST-reduced aggressive behavior. Therefore, we were not able to demonstrate a direct relationship between the SST-induced reduction in aggressive behavior and the SST-induced change in DA metabolism.

## 5 Conclusion

The current study demonstrated that administration of SST attenuates aggressive behavior in SI-reared mice. Increased HVA levels and prevention of COMT mRNA expression by SST treatment may be implicated in the anti-aggressive effects of SST. In conclusion, SST may be useful for treatment of aggressive behavior in patients with neurotic symptoms.

## Declaration of competing interest

Katsunori Iwasaki received a research grant from Tsumura & Co. (#150532).
